# Advanced low grade serous ovarian cancer: A retrospective analysis of surgical and chemotherapeutic management in two high volume oncological centers

**DOI:** 10.3389/fonc.2022.970918

**Published:** 2022-09-27

**Authors:** Paolo Di Lorenzo, Vincenza Conteduca, Emanuela Scarpi, Marco Adorni, Francesco Multinu, Annalisa Garbi, Ilaria Betella, Tommaso Grassi, Tommaso Bianchi, Giampaolo Di Martino, Andrea Amadori, Paolo Maniglio, Isabella Strada, Silvestro Carinelli, Marta Jaconi, Giovanni Aletti, Vanna Zanagnolo, Angelo Maggioni, Luca Savelli, Ugo De Giorgi, Fabio Landoni, Nicoletta Colombo, Robert Fruscio

**Affiliations:** ^1^ Obstetrics and Gynecology Unit, Morgagni-Pierantoni Hospital, Forlì, Italy; ^2^ Clinic of Obstetrics and Gynecology, San Gerardo Hospital, University of Milano-Bicocca, Monza, Italy; ^3^ Department of Medical Oncology, Istituto di ricovero e cura a carattere scientifico (IRCCS) Istituto Romagnolo per lo Studio dei Tumori “Dino Amadori”, Meldola, Italy; ^4^ Unit of Medical Oncology and Biomolecular Therapy, Department of Medical and Surgical Sciences, University of Foggia, Policlinico Riuniti, Foggia, Italy; ^5^ Biostatistics and Clinical Trials Unit, Istituto di ricovero e cura a carattere scientifico (IRCCS) Istituto Romagnolo per lo Studio dei Tumori “Dino Amadori”, Meldola, Italy; ^6^ Division of Gynecologic Oncology, European Institute of Oncology, Istituto di ricovero e cura a carattere scientifico (IRCCS), Milano, Italy; ^7^ Department of Pathology, European Institute of Oncology, Istituto di ricovero e cura a carattere scientifico (IRCCS), Milano, Italy; ^8^ Department of Pathology, San Gerardo Hospital, University of Milano-Bicocca, Monza, Italy; ^9^ Department of Hemato-Oncology, University of Milan, Milano, Italy; ^10^ Department of Medicine and Surgery, University of Milano-Bicocca, Monza, Italy

**Keywords:** low-grade serous ovarian cancer, primary cytoreduction, neoadjuvant chemotherapy, residual disease, adjuvant treatment, secondary cytoreductive surgery

## Abstract

**Simple summary:**

Low-grade serous ovarian cancer (LGSOC) represents an uncommon histotype of serous ovarian cancer (accounting for approximately 5% of all ovarian cancer) with a distinct behavior compared to its high-grade serous counterpart, characterized by a better prognosis and low response rate to chemotherapeutic agents. Similar to high-grade serous ovarian cancer, cytoreductive surgery is considered crucial for patient survival. This retrospective study aimed to analyze the outcomes of women affected by advanced stages (III–IV FIGO) of LGSOC from two high-volume oncological centers for ovarian neoplasm. In particular, we sought to evaluate the impact on survival outcomes of optimal cytoreductive surgery [i.e., residual disease (RD) <10 mm at the end of surgery]. The results of our work confirm the role of complete cytoreduction (i.e., no evidence of disease after surgery) in the survival of patients and even the positive prognostic role of a minimal RD (i.e., <10 mm), whenever complete cytoreduction cannot be achieved.

**Background:**

Low-grade serous ovarian cancer (LGSOC) is a rare entity with different behavior compared to high-grade serous (HGSOC). Because of its general low chemosensitivity, complete cytoreductive surgery with no residual disease is crucial in advanced stage LGSOC. We evaluated the impact of optimal cytoreduction on survival outcome both at first diagnosis and at recurrence.

**Methods:**

We retrospectively studied consecutive patients diagnosed with advanced LGSOCs who underwent cytoreductive surgery in two oncological centers from January 1994 to December 2018. Survival curves were estimated by the Kaplan–Meier method, and 95% confidence intervals (95% CI) were estimated using the Greenwood formula.

**Results:**

A total of 92 patients were included (median age was 47 years, IQR 35–64). The median overall survival (OS) was 142.3 months in patients with no residual disease (RD), 86.4 months for RD 1–10 mm and 35.2 months for RD >10 mm (p = 0.002). Progression-free survival (PFS) was inversely related to RD after primary cytoreductive surgery (RD = 0 vs RD = 1–10 mm vs RD >10 mm, p = 0.002). On multivariate analysis, RD 1–10 mm (HR = 2.30, 95% CI 1.30–4.06, p = 0.004), RD >10 mm (HR = 3.89, 95% CI 1.92–7.88, p = 0.0004), FIGO stage IV (p = 0.001), and neoadjuvant chemotherapy (NACT) (p = 0.010) were independent predictors of PFS. RD >10 mm (HR = 3.13, 95% CI 1.52–6.46, p = 0.004), FIGO stage IV (p <0.0001) and NACT (p = 0.030) were significantly associated with a lower OS.

**Conclusions:**

Optimal cytoreductive surgery improves survival outcomes in advanced stage LGSOC ***s***. When complete debulking is impossible, a RD <10 mm confers better OS compared to an RD >10 mm in this setting of patients.

## Introduction

Low-grade serous ovarian cancer (LGSOC) constitutes a small proportion of serous ovarian tumors (approximately 5%–10%) (1, 2). In 2004, the Two-Tier Grading system of the MD Anderson Pathology and Gynecologic Oncology Department (3) divided ovarian serous carcinoma into high-grade serous and low-grade serous. This classification has defined LGSOC as a separate entity with different histologic, molecular, and clinical patterns compared with high-grade serous ovarian carcinoma (HGSOC).

Women with LGSOC are typically diagnosed at a younger age compared to women with HGSOC (55.5 years vs 62.6 years) (1, 2). LGSOC is TP53 wild-type, unlike high-grade serous that is mainly characterized by TP53 mutations (4). The overall 5-year survival for patients with LGSOC is longer compared with HGSOC: the median OS was 90.8 vs 40.7 months in a retrospective analysis of 755 patients with LGSOC and more than 16,000 cases of HGSOC (2).

LGSOC can develop *de novo* from the ovarian surface epithelium or in the context of a borderline tumor (BOT). BOTs are distinguished from LGSOCs because of the absence of destructive stromal invasion (4, 5). The recurrence rate of BOT reported in the literature varies from 5% to 25%, according to the stage of disease (6); the majority relapse as borderline. In a survey of 276 women affected by BOT, a 6.8% recurrence rate as LGSOCs was reported (7). LGSOCs always recur as low-grade serous carcinoma; similar survival rates between LGSOCs and borderline tumors that recur as LGSOCs have been reported (2, 8).

The initial treatment of patients with stage IC-IV LGSOC is similar to that of HGSOC and includes surgery and platinum/taxane-based chemotherapy (9). Notably, the rate of chemoresistance of LGSOC has been proved to be higher than that of HGSOC, with a response rate reported in literature between 4% and 23% (2, 10, 11).

Due to its chemoresistance, surgery is definitely the cornerstone of the treatment of LGSOC, and the residual tumor at the end of surgery can influence the survival outcomes (12).

A previous study reported that complete cytoreduction at primary surgery results in an 85% 5-year overall survival (OS), while the 5-year OS rate is 32% in patients with residual disease (RD) >1 cm at the end of primary surgery (11). In the setting of recurrent LGSOC, most patients are treated with secondary cytoreductive surgery, chemotherapy, and/or hormonal therapy.

The study aims to evaluate the impact of cytoreductive surgery on survival outcomes of patients with advanced LGSOCs treated at two high-volume centers for the care of gynecologic malignancies. In particular, we assessed the effect on survival outcomes of optimal cytoreduction both at first diagnosis and at recurrence.

## Materials and methods

### Study population

This study was a retrospective analysis of ninety-two consecutive LGSOC advanced stage cases treated at Monza San Gerardo Hospital and at the European Institute of Oncology in Milan from January 1994 to December 2018.

Patients with advanced stage LGSOC [FIGO III–IV stage according to the International Federation of Gynecology and Obstetrics 1988 classification (13)] were identified. Selection criteria were 1) age ≥18 years; 2) stage III–IV FIGO LGSOC; 3) histological review of cases by pathologists with expertise in gynecological malignancies from one of the two centers, with confirmation of low-grade serous tumor of the ovary.

The exclusion criteria were: 1) non-serous low-grade ovarian cancer; 2) serous borderline ovarian tumor at the definitive histological examination; 3) stage I and II LGSOC; 4) deteriorated general health conditions with contraindication to surgery; and 5) informed consent refusal. The histological classification of LGSOC was based on the following criteria: 1) frank destructive invasion of the ovarian stroma (to differentiate low-grade serous carcinomas from serous border line tumors and serous border line tumors of micropapillarity variant), 2) relatively uniform round to oval nuclei with mild-to-moderate atypia and evenly distributed chromatin, and 3) no more than 12 mitoses per 10 high-power fields (3–5). Preoperative data were obtained from medical records, in particular the Eastern Cooperative Oncology Group performance status (ECOG) (14), value of CA 125 and the presence of ascites (more or less than 500 ml). Operative reports were reviewed and surgical cytoreductive procedures were recorded. We defined complete cytoreduction as no macroscopic RD, minimal in the case of nodules measuring 10 mm or less in maximum diameter and suboptimal when this diameter was greater than 10 mm (15). Patients underwent either primary cytoreductive surgery followed by adjuvant platinum/taxane-based chemotherapy or neoadjuvant chemotherapy (NACT) followed by interval debulking surgery and postoperative chemotherapy. In the recurrent setting, patients were divided into two groups based on medical (chemotherapy and/or hormonal therapy) or surgical treatment [secondary cytoreductive surgery (SCRS) followed by adjuvant chemotherapy and/or hormonal therapy]. For patients undergoing secondary cytoreduction, RD was estimated as for primary debulking surgery. The primary endpoint was the survival outcomes (PFS and OS) as a function of RD at primary cytoreductive surgery. Secondary endpoints were survival outcome (PFS and OS) in the recurrent setting.

### Statistical analysis

PFS was defined as the time between the date of primary surgery and the date of progression of disease or death, whichever came first. Patients who had not progressed were censored at the last tumor evaluation. OS was defined as the time between the date of surgery and the date of death from any cause or last follow-up. Descriptive statistics (absolute and relative frequency) were used for categorical variables, whereas median and interquartile ranges were used for continuous variables. Survival curves were estimated by the Kaplan–Meier method, and 95% confidence intervals (95% CI) were estimated using the Greenwood formula. Comparisons between survival curves were made using the log-rank test. Univariate and multivariate Cox regression models were used to investigate potential predictors of PFS and OS and to estimate hazard ratios (HRs) and their 95% CI. All p-values were two-sided and a p <0.05 was considered as statistically significant. Statistical analyses were performed using SAS 9.4 software (SAS Institute, Cary, NC, USA).

## Results

### Patients’ characteristics

During the study period, 97 patients received primary-debulking surgery or interval debulking surgery. Five patients were lost to follow up and thus excluded from the analysis. Overall, 92 patients with advanced LGSOCs were included in this study.

The median age at diagnosis was 47 years (IQR 35–64). Most patients had a good performance status (ECOG 0–1); the median preoperative serum CA-125 level was 248 U/ml (IQR 93–647). Patient demographics and clinical characteristics are described in **Table 1**. Most of the patients had stage IIIC (63%), and 19.6% were stage IV.

**Table 1:** Characteristics of Patients with stage III–IV LGSOC.

Characteristics	All patients (n = 92)
Median age (range and IQR)	47 y (20–81, 35–64)
CA 125 at diagnosis (U/ml): median value (range,IQR)	248 (15–6,186, 93–647)
Ascites at surgery	
Ascites >500 ml Ascites <500 ml No ascites Unknown	26 (28.3%) 29 (31.5%) 34 (37%) 3 (3.2%)
FIGO stage	
IIIA IIIB IIIC IVA IVB	4 (4.4%) 12 (13%) 58 (63%) 7 (7.6%) 11 (12%)
Residual disease after primary surgery	
NED 1–10 mm >10 mm Unknown	39 (42.3%) 32 (34.8%) 18 (19.6%) 3 (3.3%)

Data reported as median, numbers and percentage; y, years; NED, no evidence of disease; IQR, interquatile range.

Fourteen patients (13%) received NACT, of whom six patients (42.8%) because of high tumor burden in the abdomen with no possibility of complete tumor resection, seven patients (50%) who had already started chemotherapy when referred to one of the two centers, and one patient (7.1%) due to the diagnosis of pulmonary embolism on the staging CT scan. Six (42.9%) of fourteen patients underwent laparoscopic biopsy in one of the two centers. Seven patients (50%) had already started chemotherapy after a previous biopsy. The diagnosis was confirmed by pathologists from one of the two centers. In one woman (7.1%), who had pulmonary embolism, staging was performed on the basis of thoraco-abdomino-pelvic CT and then confirmed at final pathology after an interval of debulking surgery. According to RECIST Criteria 1.1 version (16), five patients (36%) had a partial response, three patients (21%) had stable disease, and four patients (28%) had progression of disease. Of two patients (15%) response rate was not reported according to the RECIST criteria for the medical record. Complete cytoreduction (no macroscopic residual disease) was achieved in 42.3% of the patients. In thirty-two (34.8%) patients, RD was 1–10 mm at the end of surgery. Eighteen patients (19.6%) had RD >1 cm. For three patients (3.3%), RD was not reported, but they were nevertheless included in the statistical analysis.

### Treatment procedures

Most patients underwent laparotomy (92.3%). The surgical procedures performed are reported in **Table 2**. The most common surgical procedures were hysterectomy, bilateral salpingo-oophorectomy, and omentectomy. Thirty-one (34%) patients did not undergo hysterectomy, with seventeen of them (54.8%) due to their young age and the desire to preserve the uterus. Eleven patients (35.5%) had previously undergone hysterectomy for non-cancerous reasons; in three patients (9.7%), only palliative surgery was performed due to the extent of disease with no possibility for complete cytoreductive surgery.

**Table 2:** Surgical procedures performed at primary surgery.

Surgical procedure	Number of patients (%)
Hysterectomy	61 (66%)
Oophorectomy	
Bilateral Unilateral or cystectomy	67 (72.8%) 25 (27.2%)
Omentectomy	77 (81.5%)
Lymphadenectomy	
Pelvic lymphdenectomy Pelvic and paraortic lymphadenectomy	6 (6.5%) 23 (25%)
Bowel resection	
Large bowel Small bowel Small and large bowel	26 (28.3%) 2 (2.2%) 8 (8.7%)
Diaphgragm surgery	
Stripping Resection	12 (13%) 15 (16.3%)
Splenectomy	7 (7.6%)
Stoma	5 (5.5%)

About 30% of patients also underwent bowel surgery (mainly recto-sigmoid resection) and diaphragmatic surgery (stripping or resection). In 5.5% of cases, an intestinal stoma was necessary. Splenectomy was performed in seven patients (7.5%). Eight-eight patients (95.6%) received adjuvant therapy after surgery: in detail seventy-three patients (83%) received chemotherapy, six patients (6.8%) hormonal therapy, nine patients (10.2%) both chemotherapy and hormonal therapy. Four patients (4.4%) did not receive adjuvant therapy (one died of disease progression before starting chemotherapy, one patient declined chemotherapy after discharge, and two patients did not receive the adjuvant treatment for severe comorbidities and old age).

Among patients who received adjuvant chemotherapy, forty-four (53.7%) received carboplatin and paclitaxel; eleven (13.4%) received carboplatin and paclitaxel in association with bevacizumab; carboplatin in monotherapy was used in seventeen patients (20.7%). Seven patients (8.5%) received different chemotherapeutic regimens, such as liposomal doxorubicin, carboplatin and gemcitabine, carboplatin and paclitaxel ± placebo/BIBF (study s517/609) (17), carboplatin and gemcitabine, liposomal doxorubicin plus mTOR inhibitor in an experimental study, cyclophosphamide, methotrexate and fluorouracil, cyclophosphamide, adriamycin and platinum. There are no data about the regimens of adjuvant treatment for three patients (3.7%).

Sixty patients received six cycles (73.2%), four patients (4.9%) three cycles, three patients (3.6%) five cycles, three patients (3.6%) eight cycles, one patient (1.2%) nine cycles, one patient (1.2%) seven cycles, one patient (1.2%) four cycles and one patient (1.2%) one cycle of experimental therapy with mtor inhibitor, then interrupted for progression disease; for eight patients (9.8%) number of cycles was unknown.

In the NACT setting, nine patients (64.2%) received three cycles of chemotherapy, two patients (14.2%) two cycles, and three patients (21.4%) six cycles. Carboplatin and paclitaxel was administered in thirteen patients (92.9%). In one patient (7.1), docetaxel was given due to an allergic reaction to paclitaxel after the second cycle. All patients treated with NACT also received adjuvant post-operative treatment after surgery (eight received chemotherapy, six hormonal therapy).

Estrogen and progesterone profiles were unknown in 85.9% and 89.1% of patients, respectively. In thirteen patients (14.1%) of whom estrogen receptors were searched, twelve (92.3%) tested positive. Progesterone was known in ten patients (10.9%); of these, six (60%) showed receptor expression. As regards hormonal therapy, tamoxifen was prescribed in ten patients (66.7%), anastrozole was used in four patients (26.7%), and letrozole in one patient (6.7%).

In the cohort of eighteen patients with residual disease more than 10 mm, sixteen (88.9%) received adjuvant treatment: thirteen patients (81.2%) were administered with chemotherapy; two patients (12.5%) received both chemo and hormonal therapy, and one patient (6.2%) received hormonal therapy alone. In this subgroup with large-post-operative residual disease, according to the RECIST Criteria 1.1 version (16), Four patients (25%) had a complete or partial response; one patient (6.2%) had stable disease; three patients (18.8%) had a progression of disease and in eight patients (50%) it was impossible to set up a response rate from medical records.

### Clinical outcomes

The median follow-up was 116 months (range 1–377). Seventy-four patients (80.4%) had a recurrence; the median PFS was 25.1 months (95% CI 15.2–32.2) for all patients. The median OS was 86.4 months (95% CI: 52.3–142.3). RD at the end of surgery was associated with better PFS, with a median PFS of 38.3 months (95% CI 16.2–57.3) in patients with no RD vs 23.3 months (95% CI 12.0–32.6) for patients with RD 1–10 mm and 14.8 months (95% CI 2.5–27.5) for patients with RD >10 mm (p = 0.002). Similar results were observed for OS: 142.3 months (95% CI 48.8–not reached) for patients with no RD vs 86.4 months (95% CI 54.1–163.3) for patients with RD 1–10 mm and 35.2 months (95% CI 15.6–49.9) for patients with RD >1 cm (p = 0.002).

**Figure 1:**
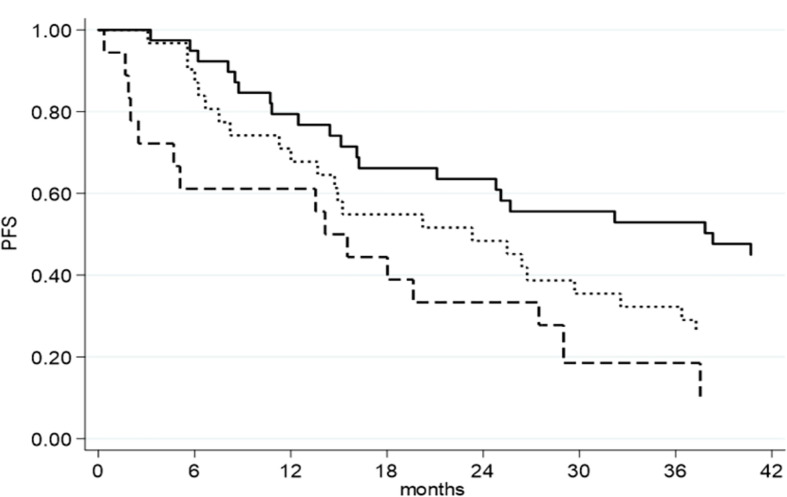
PFS as function of residual disease at primary cytoreductive surgery. 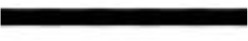 RD= NED; 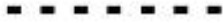 RD= 1-10 mm; 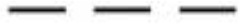 RD= >10 mm.

The univariate analysis of PFS showed that RD between 1–10 mm (HR = 1.82, 95% CI 1.06–3.11, p = 0.027), RD >10 mm (HR = 3.04, 95% CI 1.59–5.83, p = 0.003), and FIGO stage IV (HR = 1.86, 95% CI 1.06–3.27, p = 0.031) were associated with an increased risk of recurrence. On multivariate analysis, RD between 1–10 mm (HR = 2.30, 95% CI 1.30–4.06, p = 0.004), RD >10 mm (HR = 3.89, 95% CI 1.92–7.88, p = 0.0004) (**Figure 1**), and FIGO stage IV (HR = 2.83, 95% CI 1.52–5.26, p = 0.001) were significantly associated with worse PFS. We observed that NACT correlated with shorter PFS both at univariate (HR = 2.09, 95% CI 1.12–3.91, p = 0.021) and multivariate analysis (HR = 2.33, 95% CI 1.23–4.84, p = 0.010) (**Table 3**).

**Table 3:** Factors predicting Progression Free Survival.

Variables	Univariate analysis		Multivariate analysis	
	HR (95% CI)	p-value	HR (95% CI)	p-value
Age	1.015 (0.99–1.02)	0.556	–	–
ECOG PS	2.04 (0.87–4.79)	0.100	–	–
CA 125 (>35 vs <35)	1.50 (0.54–4.3)	0.436	–	–
Neoadjuvant chemotherapy	2.09 (1.12–3.91)	0.021	2.33 (1.23–4.84)	0.010
Adjuvant therapy	0.29 (0.09–0.94)	0.038	0.24 (0.07–0.86)	0.029
FIGO stage (IV vs III)	1.86 (1.06–3.27)	0.031	2.83 (1.52–5.26)	0.001
Residual disease (1–10 mm vs NED)	1.82 (1.06–3.11)	0.027	2.30 (1.30–4.06)	0.004
Residual disease (>10 mm vs NED)	3.04 (1.59–5.83)	0.003	3.89 (1.92–7.88)	0.0004

**Figure 2:**
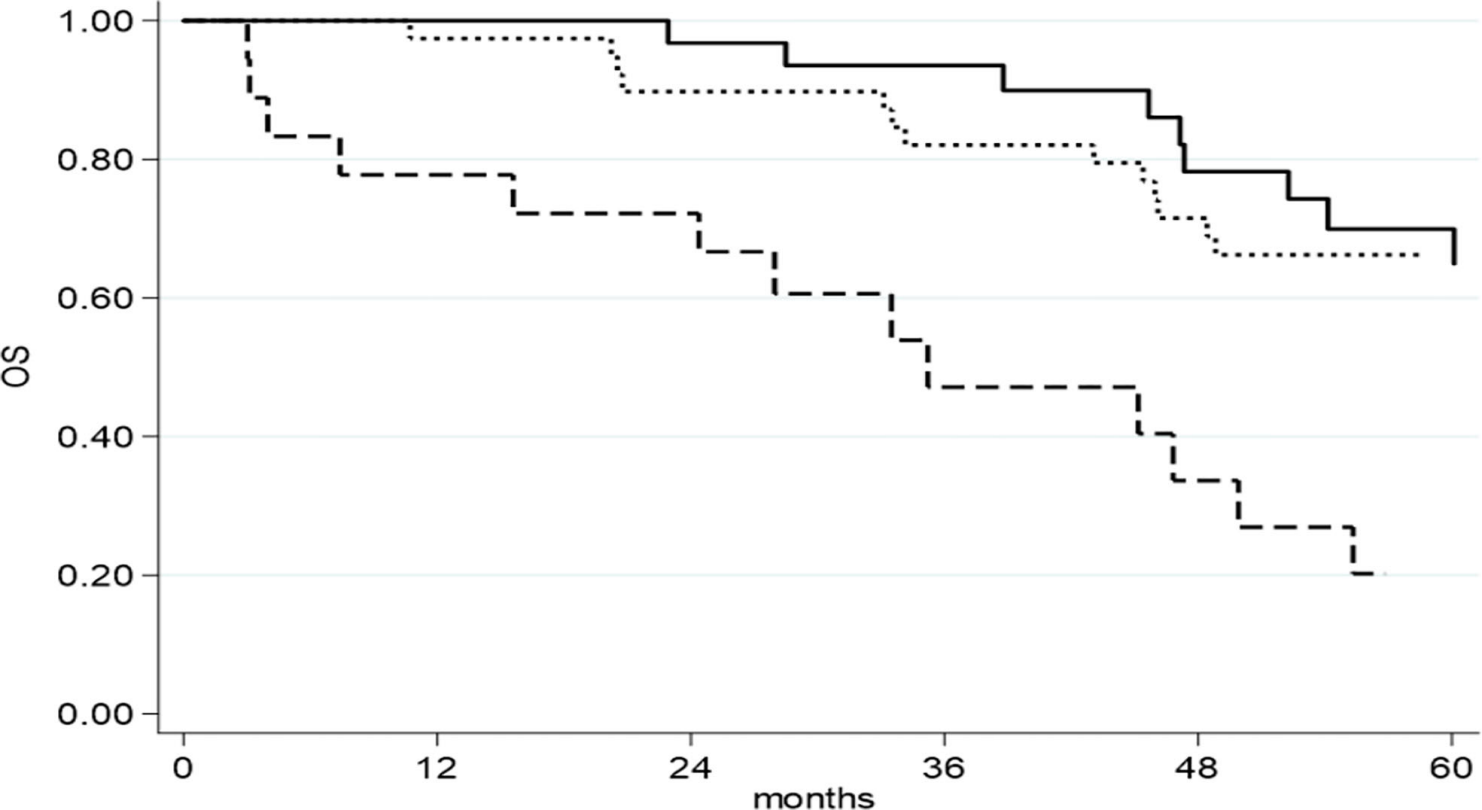
OS as function of residual disease at primary cytoreductive surgery. 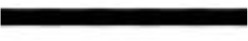 RD= NED; 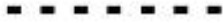 RD= 1-10 mm; 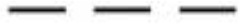 RD= >10 mm.

Considering OS, at univariate analysis, RD >10 mm (HR = 3.13, 95% CI: 1.52–6.46, p = 0.004), FIGO IV stage (HR = 3.12, 95% CI: 1.61–6.04, p = 0.0007), ECOG PS (HR = 3.75, 95% CI: 1.55–9.03, p = 0.003) and NACT (HR = 2.28, 95% CI: 1.15–4.53, p = 0.019) were associated with worse survival. On multivariate analysis, RD >10 mm (HR = 3.29, 95% CI: 1.50–7.24, p = 0.007) (**Figure 2**), FIGO stage IV (HR = 4.40, 95% CI: 2.09–9.27, p <0.0001) and NACT (HR = 2.24, 95% CI: 1.08–4.66, p = 0.030) were maintained as statistically significant (**Table 4**).

**Table 4:** Factors predicting Overall Survival.

Variables	Univariate analysis		Multivariate analysis	
	HR	p-value	HR	p-value
Age (yr)	1.02 (0.99–1.04)	0.057	1.09 (0.45–2.60)	0.853
ECOG PS	3.75 (1.55–9.03)	0.003	2.48 (0.70–8.80)	0.160
CA 125 (>35 vs <35)	1.05 (0.37–2.98)	0.924	–	–
Neoadjuvant chemotherapy	2.28 (1.15–4.53)	0.019	2.24 (1.08–4.66)	0.030
Adjuvant therapy	0.48 (0.15–1.56)	0.223	0.49 (0.14–1.72)	0.264
FIGO stage (IV vs III)	3.12 (1.61–6.04)	0.0007	4.40 (2.09–9.27)	<0.0001
Residual disease (1–10 mm vs NED)	1.07 (0.54–2.11)	–	1.18 (0.57–2.42)	–
Residual disease (>10 mm vs NED)	3.13 (1.52–6.46)	0.004	3.29 (1.50–7.24)	0.007

In the recurrent population (84 patients), forty-nine patients (66.2%) received chemotherapy or hormonal therapy, and twenty-five (33.8%) underwent secondary cytoreductive surgery (SCRS). In analyzing survival outcomes of patients treated with surgery at recurrence, we excluded three patients (one underwent neurosurgical operation for brain metastasis; one patient underwent second-look surgery, that was not considered as SCRS; and one case with inadequate information in medical records about SCRS).

Thirteen patients (59%) had no macroscopic RD at the end of SCRS, two patients (9%) had RD between 1–10 mm, and seven patients (32%) had RD >10 mm.

The median PFS from SCRS was 18 months (95% CI: 4.6–32.7) and the median OS was 63.3 months (95% CI: 27.1–130.7). Despite the small number of patients and events, our results indicated a slight, albeit not significant, benefit in OS for patients undergoing SCRS without macroscopic RD compared with SCRS with >10 mm of tumor (HR = 8.54, 95% CI: 0.71–102.73; p = 0.068) (**Tables 5**, **6**).

**Table 5:** Factors predicting Progression Free Survival in recurrent population undergoing SCRS.

Variables	Univariate analysis		Multivariate analysis	
	HR	p-value	HR	p-value
FIGO stage of disease (IV vs III)	2.01 (0.54–7.45)	0.296	15.08 (1.93–117.99)	0.010
RD primary cytoreductive surgery				
1–10 mm vs NED	0.37 (0.10–1.32)		0.25 (0.06–1.03)	
>10 mm vs NED	0.24 (0.05–1.18)	0.121	0.08 (0.01–0.063)	0.027
RD secondary cytoreductive surgery				
1–10 mm vs NED	3.16 (0.37–26.70)	–	3.83 (0.42–35.33)	–
>10 mm vs NED	1.86 (0.69–5.02)	0.342	3.48 (1.14–10.59)	0.067

**Table 6:** Factors predicting Overall Survival in recurrent population undergoing SCRS.

Variables	Univariate analysis		Multivariate analysis	
	HR	p-value	HR	p-value
FIGO stage of disease (IV vs III)	4.04 (0.94–17.40)	0.061	1.54 (0.10–24.10)	0.759
RD primary cytoreductive surgery				
1–10 mm vs NED >10 mm vs NED	1.37 (0.24–7.88) 3.01 (0.69–13.17)	- 0.325	1.62 (0.19–13.56) 3.53 (0.43–29.02)	– 0.491
RD secondary cytoreductive surgery				
1–10 mm vs NED >10 mm vs NED	ne 24.76 (2.71–225.79)	– 0.004	28.95 (1.42–590.23) 8.54 (0.71–102.73)	– 0.068

Ne, not estimable.

## Discussion

The present multi-center study represents one of the largest reports of advanced LGSOCs, a rare form of ovarian neoplasm. In the AGO (Arbeitsgemeinschaft Gynäcologische Onkologie) group database, out of 5114 patients with ovarian cancer, only 145 (2.8%) had an advanced stage of LGSOC (9).

Previous studies demonstrated residual disease at the end of primary cytoreductive surgery to be the most important factor influencing survival in advanced LGSOCs (11, 18). Gershenson et al. reported a worse survival in patients with persistent disease after primary cytoreduction and adjuvant chemotherapy (18). In a more recent AGO group analysis, 5-year OS in patients with complete cytoreduction was 85% vs 32% of patients with residual tumor >10 mm (p <0.001); nearly complete resection with RD up to 10 mm showed benefit with 5 year OS of around 61% (11).

Our data confirm that complete cytoreduction correlates with an improvement of survival outcomes, not only in terms of PFS but also of OS. This evidence underlines that surgery is the most important therapeutic weapon in patients with advanced LGSOC. Historically, LGSOC was not considered a chemoresponsive disease. Schmeler et al. (19) showed a 4% complete response and an 88% stable disease rate in 25 patients undergoing NACT. Grabowski et al. (11) reported a response rate of 23.1% in 39 patients with RD greater than 10 mm after primary cytoreductive surgery. Prior studies showed that a chemotherapy response rate higher than 25% is not documented in LGSOC (20). These data are reinforced by the observation that interval debulking surgery after NACT seems not to be effective, indicating a marginal role for chemotherapy in treating these patients. Bogani et al. (21) demonstrated a shorter survival in patients with LGSOC who received NACT followed by interval debulking surgery, compared to patients undergoing upfront cytoreductive surgery. In a comparison study between LGSOC and HGSOC patients treated with NACT, Cobb et al. (22) reported 11% of partial response, 83% of stable disease, and 6% of progressive disease in thirty-six patients with LGSOC, while in thirty-six HGSOC patients, they observed 75% of partial response and 25% of stable disease. This confirms the different sensitivity to chemotherapy of LGSOC compared to the high grade histology and reinforces doubts about the use of this therapeutic approach. However, the same authors suggest that NACT should not be abandoned. In our study, patients undergoing NACT had worse survival. Nevertheless we do not feel like stating that NACT has a detrimental effect because this could be a bias due to the selection of patients unfit for cytoreductive surgery at diagnosis and so with worse prognosis. Despite this, our response rate in NACT was 36%, higher than in other previous published studies.

In the adjuvant setting, our response rate, considering complete and partial responses, was 25%, similar to data reported by Grabowski et al. (11).

Given these preliminary considerations, and highlighting the paramount importance of surgery in the treatment of LGSOC, the absence of macroscopic RD is the most important therapeutic goal. In our series, patients with RD >10 mm have a worse prognosis compared to RD 1–10 mm and RD = 0 mm. While RD 1–10 mm in comparison to RD 0 mm increased the risk of recurrence at multivariate analysis, we did not observe a worse OS in patients with RD 1–10 mm compared to RD = 0 mm. Such result may suggest the possibility to module aggressive cytoreductive surgery aiming toward a RD as minimal as possibile, when RD = 0 cannot reach.

For the recurrent disease, we only analyzed patients who were treated with secondary cytoreductive surgery. Because of the small number of patients included in this setting, there is only a trend towards better OS after complete cytoreduction. This would be in line with a study of the MD Anderson group (23) which reported an advantage both in PFS (p = 0.008) and OS (p = 0.04) for patients with RD = 0 mm at secondary cytoreduction. This work suggests that patients undergoing direct secondary cytoreduction at the time of recurrence have a better OS compared to women receiving chemotherapy for recurrence before secondary surgery. The role of surgery in recurrent LGSOC could be important as the chemotherapy response rate in this setting is even lower than in first line; Gershenson et al. (24) reported a response rate of 3.7% in 58 patients with recurrent LGSOC.

The chemoresistance of LGSOC is the most important factor that hampers the treatment of LGSOC women. Recently, Gershenson et al. (25) demonstrated the importance of hormonal maintenance therapy (HMT) after primary treatment with surgery and adjuvant chemotherapy in patients with LGSOC. In this study, women who received hormonal therapy had a significantly longer PFS compared with women who underwent routine follow-up, both for those who were disease free and those who had persistent disease (81.1 vs 30 months, p = 0.001 and 38.1 vs 15.2 months, p = 0.001). A lower risk of progression was reported in patients receiving HMT compared to the observational group (HR = 0.44, 95% CI: 0.31–0.64, p <0.001).

In patients with a recurrence due to chemoresistance of common chemotherapeutic drugs, bevacizumab could have a role. Dalton et al. (26) reported a 40% partial response and 30% stable disease in the cohort of women with recurrent LGSOC treated with a combination of chemotherapy plus bevacizumab; Grisham et al. (27) described a 40% overall response rate in 15 patients with recurrent LGSOC of the ovary and peritoneum.

A novel and recent therapeutic approach, taking into consideration the high frequency of KRAS and BRAF mutations in LGSOC (4, 5, 28, 29) and the activation of the MEK–MAPK pathway (30), is the administration of MEK inhibitors. Different trials have explored the activity of these drugs in recurrent LGSOC (31–33), finding an overall response rate (ORR) between 15% and 26% with three different MEK inhibitors [selumetinib (31), binimetinib (32), and trametinib (33)]. Hormonal therapy may also be active in the recurrence setting. A phase II study (34) regarding anastrozole activity in patients with estrogen receptors-positive recurrent or metastatic LGSOC (PARAGON study) showed 14% partial response and 50% stable disease, with a low rate of adverse events. Expression of the estrogen receptor (ER) and progesterone receptor (PR) could be important for predicting response to hormonal therapy. A recent metaanalysis analyzed 437 cases of LGSOC, reporting expression of ER in 80.7% and PR in 54.4% (35). A correlation between hormone receptor expression and survival outcome has been demonstrated. Sehouli et al. reported a statistically significant correlation between the percentage of expression of ER and PR and PFS. For OS authors observed a tendency towards better OS for LGSOC expressing hormonal receptors (36).

The limitations of the present study are the retrospective design; lack of data in a few patients; and different treatments regarding both surgery (primary and interval debulking surgery) and medical treatment (chemotherapeutic and hormonal). It must be pointed out that patients with more advanced disease could have a higher chance of having neoadjuvant chemotherapy, and this might be a bias when evaluating the effectiveness of NACT. In fact, these patients may show a worse outcome compared to those who received primary debulking surgery. Therefore, it is not possible to draw any conclusion regarding the value of NACT in these patients. Moreover, it should be noted that, even if patients were treated in two referral centers for gynecologic oncology with excellent surgical expertise, different surgeons operated on them in the study period and this might have impacted on surgical outcome; we calculated a total of ten surgeons across the two institutions during the study period.

The main strengths are the large sample size, the long follow-up, the review of the pathological specimens by pathologists with special expertise in gynecological malignancies, and the surgical treatment in two high-volume centers for gynecological oncology.

## Conclusion

In conclusion, our findings underline that primary cytoreductive surgery with RD <10 mm in advanced LGSOC ensures better PFS and OS in comparison with suboptimal operation (RD >10 mm). Despite the fact that cytoreduction with no macroscopic RD would be the target of surgery, our study reveals an important advantage both in terms of OS and PFS for patients with minimal residuals up to 10 mm in comparison to patients with more than 10 mm of tumor at primary operation.

## Data availability statement

The original contributions presented in the study are included in the article/supplementary material. Further inquiries can be directed to the corresponding authors.

## Ethics statement

Ethical review and approval was not required for the study on human participants in accordance with the local legislation and institutional requirements. The patients/participants provided their written informed consent to participate in this study.

## Author contributions

PD was involved in the conception of the study, acquisition and analysis of the data, and wrote the first draft of the manuscript. PDL, MA, AG, IB, TG, GDM, TB, IS, SC, MJ, GA, VZ, and AM were involved in the acquisition of the data. PDL, VC, ES, MA, FM, NC, FL, and RF were involved in the conception and design of the study. PDL, VC, ES, FM, AA, PM, LS, UD, NC, FL, and RF contributed to data analysis and interpretation of data. VC, ES, FM, FL, NC, and RF critically revised the manuscript for important intellectual content. PDL, VC, ES, NC, FL, and RF participated in analyzing the results and drafting the manuscript. All authors contributed to the article and approved the submitted version.

## Conflict of interest

NC: Consultancy and advisory board participation: Roche; PharmaMar; Astra-Zeneca; Clovis Oncology; MSD; GlaxoSmithKline; Tesaro; Pfizer; BIOCAD; Immunogen; Mersana; Eisai; Oncxerna. Speakers for AstraZeneca, Tesaro, Novartis, Clovis, MSD, GlaxoSmithKline, Eisai.

The remaining authors declare that the research was conducted in the absence of any commercial or financial relationships that could be construed as a potential conflict of interest.

## Publisher’s note

All claims expressed in this article are solely those of the authors and do not necessarily represent those of their affiliated organizations, or those of the publisher, the editors and the reviewers. Any product that may be evaluated in this article, or claim that may be made by its manufacturer, is not guaranteed or endorsed by the publisher.
